# Step Length Measurements Using the Received Signal Strength Indicator

**DOI:** 10.3390/s21020382

**Published:** 2021-01-07

**Authors:** Zanru Yang, Le Chung Tran, Farzad Safaei

**Affiliations:** School of Electrical, Computer and Telecommunications Engineering, University of Wollongong, Wollongong, NSW 2522, Australia; lctran@uow.edu.au (L.C.T.); farzad@uow.edu.au (F.S.)

**Keywords:** wireless body area networks, step length, gait speed, path loss, non-GPS localization, distance measurement

## Abstract

In this paper, portable transceivers with micro-controllers and radio frequency modules are developed to measure the received signal strength, path loss, and thus the distance between the human ankles for both indoor and outdoor environments. By comparing the experimental results and the theoretical model, a path loss model between transceivers attached to the subject’s ankles is derived. With the developed experimental path loss model, the step length can be measured relatively accurately, despite the imperfections of hardware devices, with the distance errors of a centimeter level. This paper, therefore, helps address the need for a distance measurement method that has fewer health concerns, is accurate, and is less affected by occlusions and confined spaces. Our findings possibly lay a foundation for some important applications, such as the measurement of gait speed and localization of the human body parts, in wireless body area networks.

## 1. Introduction

Wireless body area networks (WBANs) are radio networks of sensors placed on, in, around, or near the human body, which represent the latest generation of personal area networks [1]. There is a wide range of applications of WBANs, including medical treatments, healthcare, sports, emergency services, and military [2]. To implement the above applications, accurate localization algorithms are required, whose core task is usually the distance measurement. However, compared to other wireless sensor networks (WSNs), the wireless channels of WBANs show a variety of characteristics due to the presence of human body and the complex surrounding environments [3,4]. Additionally, some peculiar factors, including antenna losses, energy absorption, and shadowing effect, caused by body tissues and postures will result in a significant signal attenuation.

As a well-known localization positioning system for outdoor, GPS may fail to meet the accuracy requirements for some indoor environments, because the electromagnetic wave is severely attenuated by obstacles or is even forbidden in some situations, such as in military operations. Therefore, non-GPS localization has gained much interest from both researchers and industries [5]. Range-free and range-based are two categories of non-GPS localization. Range-free methods use signals to define a region that contains the unknown node whereas range-based methods require additional hardware to assist the estimation [6]. Each of these methods has its merits and demerits. Range-free methods are simple and energy-efficient, but the accuracy is low [7]. Based on the additional information provided by hardware, such as angles or distances, range-based techniques are capable of estimating the angles and distances between the unknown nodes and the anchor nodes with a higher accuracy [8] than the range-free ones, especially in a noisy environment.

Therefore, this paper focuses on the range-based methods. The method of infrared thermography was applied in Reference [9] to detect the gait patterns of human and achieved an accuracy of 78–91%. However, due to the used of laser requires well-trained staff, the pricey equipment, and the potential harm for long time exposure, it is not an ideal method for the step length measurement in a daily basis. The ultrasonic technique measures the time sound required to send and receive the reflected wave from the target object, which can then be transferred to the distance between two points [10]. Although the ultrasonic ranging system is accurate with the errors in the 10-cm level, it needs expensive hardware and is affected by temperature and humidity [11]. Another representative in the range-based methods is the Received Signal Strength Indicator (RSSI), which features a low communication overhead and low complexity. The RSSI can be used for a double purpose of distance measurement and node localization technology. It has advantages, including simple and inexpensive hardware. The RSSI technique relies on the signal strength of the received signal, which is a function readily available or simple to obtain using most commercial transceivers [11].

Table 1 compares several RSSI-based localization and distance estimation techniques in terms of channel model, hardware, measured data set, as well as the experimental range and accuracy. For example, researchers used the ZigBee-based hardware platform to analyze the RSSI measurement error in Reference [12] with a data set of 100 samples in each experiment. In a range of 20 m, the error is as large as 2 m, so it is not able to localize the node correctly. In Reference [13], the distance was estimated based on the ZigBee hardware and the Maximum Likelihood (ML) detection method. With a sensor node density of 0.27 nodes/m${}^{2}$, the estimation error can be reduced to 1.5 m in a 7.08 m × 10.60 m conference room. However, both of the above researches have collected a limited number of RSSI values, which may affect their confidence in the estimated accuracy. In Reference [14], the accuracy of the RSSI-based algorithm is improved with WiFi access points. Because usually the locations of these access points are fixed in a building, candidates in the building can estimate their position by the trilateration method, which requires at least three known WiFi access points. In Reference [15], both multilateration and trilateration algorithms were used to measure the distance between the anchors and the target node based on the RSSI values. Comparing to the single antenna system, the measurement accuracy is improved by applying multiple antennas at both transmitter and receiver. Authors in [16,17] also proposed a multi-antenna system which equally transmitted the power at a low peak-to-average power ratio (PARP). RSSI-based Bluetooth positioning was analyzed in Reference [18]. The authors proposed an experimental model for the indoor propagation and used triangulation to measure the distance between two Bluetooth devices. The positioning system yielded good results for an indoor environment but also showed about 6–8 dB attenuation when Bluetooth devices, such as mobile phones, are covered by the human body. Therefore, shadowing effect is a main challenge in the RSSI method.

In Reference [19], the authors discussed the relationship between RSSI variation and different directions of the antenna in a walking scenario when the hardware was attached to the human’s wrists. The change in the relative positions of the human’s wrists with respect to the human’s torso causes different shadowing levels. Authors in Reference [20] improved the accuracy and self-adaptability of the log-normal shadowing model (LNSM) by applying a log-normal shadowing model with the dynamic variance (LNSM-DV) and least square (LS) method. In Reference [21], the authors combined RSSI and Angles-of-Arrival (AOA) to estimate the position of an unknown node. This hybrid RSSI-AOA estimation algorithm collected different RSSIs for different angles by rotating the node at the same place. The strength of the received signal indicates the main lobes of the radiation pattern and thus the zone of the unknown node. Similarly, the authors in References [22,23] rotated the antenna direction and then recorded the received power at different angles. By finding the strongest signal from the received power spectrum, the position of the unknown node can be identified. A weighted combination methods of RSSI and AOA was explored in Reference [5]. The observation indicates that the RSSI component is more sensitive to shadowing than the AOA component in vehicular ad hoc network (VANET) scenarios. In the simulation, the accuracy of correctly localizing the unknown node was claimed to be 83%. However, a thorough analysis of estimating the distance between nodes on human bodies in daily life activities is still missing. Therefore, it is worth to explore the performance of the RSSI-based method in distance measurement in WBANs with different hardware configurations.

In this paper, we focus on the distance measurement between two human body parts in WBANs scenarios using the hardware-based RSSI approach. Inspired by our previous related work [2,24,25], we first develop the transceivers and use them to measure their distance when they are attached to the human ankles. We then propose an experimental path loss model between the two ankles of the subject under test, that jointly considers the path loss, shadowing effect, multipath, mismatch loss, and insertion loss. The static scenario where the subject under test stands still with different step lengths is considered to explore the characteristics of the wireless channel between the two ankles. We will demonstrate the channel between two ankles follows an empirical model, which is the free space model added with a correction factor ΔPL. Different configurations are taken into consideration to find the most suitable transmission power level and data rate for the distance measurements.

The main contributions of this paper are as follows.

- Derivation of an empirical path loss model between two transceivers attached to the human ankles. The proposed model jointly considers the path loss, hardware non-linearity, shadowing effect, multipath, mismatch loss, and insertion loss.
- Estimation and accuracy analyses of the distance estimation between the transmitter and the receiver based on the proposed path loss model between the two ankles.

The rest of the paper is organized as follows. Section 2 describes the system model. The experimental materials are detailed in Section 3, and the newly proposed path loss model are presented in Section 4. The experiment outcomes and analysis are detailed in Section 5. Section 6 concludes the paper. Finally, Section 7 states the limitations and the future research directions of this work.

## 2. System Model

There are several radio propagation models to predict the received signal power, including the free-space propagation, two-ray ground reflection, and IEEE 802.15.6 path loss models. The two latter models are not applicable in this paper because they are not suitable with the experiment settings in this paper. More specifically, the experiments in this paper are carried out for distances no longer than one meter between the two human ankles in the realistic environment, rather than for elevated antennas over a several kilometer distance or for the propagation environment in an anechoic chamber [3]. Meanwhile, the free space model is close to the propagation environment in our experimental settings but requiring an adaptation via the correction factor ΔPL.

In this paper, we estimate the distance between the transmitter and the receiver within the range of a human step length, i.e., no more than one meter in both indoor and outdoor environments. The transmitter and the receiver are attached to the ankles of the subject under test in a way that the antennas face each other. This means there exists a Line-Of-Sight (LOS) path between the transceivers. As a result, the experimental path loss, denoted as $PL_{OA}$, is conjectured to be the free space path loss, denoted as $PL_{FS}$, plus a correction factor ΔPL, which covers some extra losses, including shadowing effect, multipath, mismatch loss, and insertion loss, i.e.,

(1) $$PL_{OA}\left(dB\right)=PL_{FS}+\Delta PL.$$

The free space path loss, $PL_{FS}$, is derived from the Friis transmission equation [26], which states that the ratio of the received power $P_{r}$ to the transmitted power $P_{t}$ is

(2) $$\frac{P_{r}}{P_{t}}=\frac{G_{t}G_{r}\lambda^{2}}{{\left(4\pi d\right)}^{2}},$$

where $G_{t}$ and $G_{r}$ are the antenna gains of the transmitter and the receiver, respectively; λ is the wavelength of the signal, and *d* is the distance between the antennas. Equation (2) requires the receive antenna to be located at the far field of the transmit antenna. The free space path loss is defined as

(3) $$PL_{FS}={\left(\frac{4\pi d}{\lambda }\right)}^{2}.$$

From Equation (3), path loss can also be presented in dB as

(4) $$PL_{FS}\left(dB\right)=20\log_{10}\left(f\right)+20\log_{10}\left(d\right)-27.56,$$

where *f* is the radio frequency in MHz, and *d* is the distance between the transmit and receive antennas in meters. This formula will be used to calculate the theoretical free space path loss, which is used as a benchmark in our experiments. The path loss between two transceivers can be expressed in dB as

(5) $$\begin{array}{cc} PL_{OA}\left(dB\right) & =P_{t}-P_{r}+G_{t}+G_{r}\\ \; & =P_{t}+RSSI, \end{array}$$

where $RSSI\left(dB\right)=|-P_{r}+G_{t}+G_{r}|$ with a note that $P_{r}$ (dB) is a negative value.

## 3. Materials and Data Collection Setup

In this section, we will detail the materials required for the experiments from both software and hardware aspects.

Arduino Integrated Development Environment (IDE) and XBee Configuration & Test Unit (X-CTU) are the software used in this project. They are used to program the Arduino UNO microprocessors and to configure parameters of the XBee-PRO S2C wireless transceivers. When programming the Arduino UNO hardware using Arduino IDE, the baud rate must be declared to be the same as that of the transmitting and receiving Xbee-PRO S2C modules. These Xbee-PRO S2C modules are configured by X-CTU. In X-CTU, apart from the data rate and the power level, we should also configure the addresses of the source and destination, pair them, set the transmitter as a coordinator, and the receiver as an end device.

**Figure 1:**
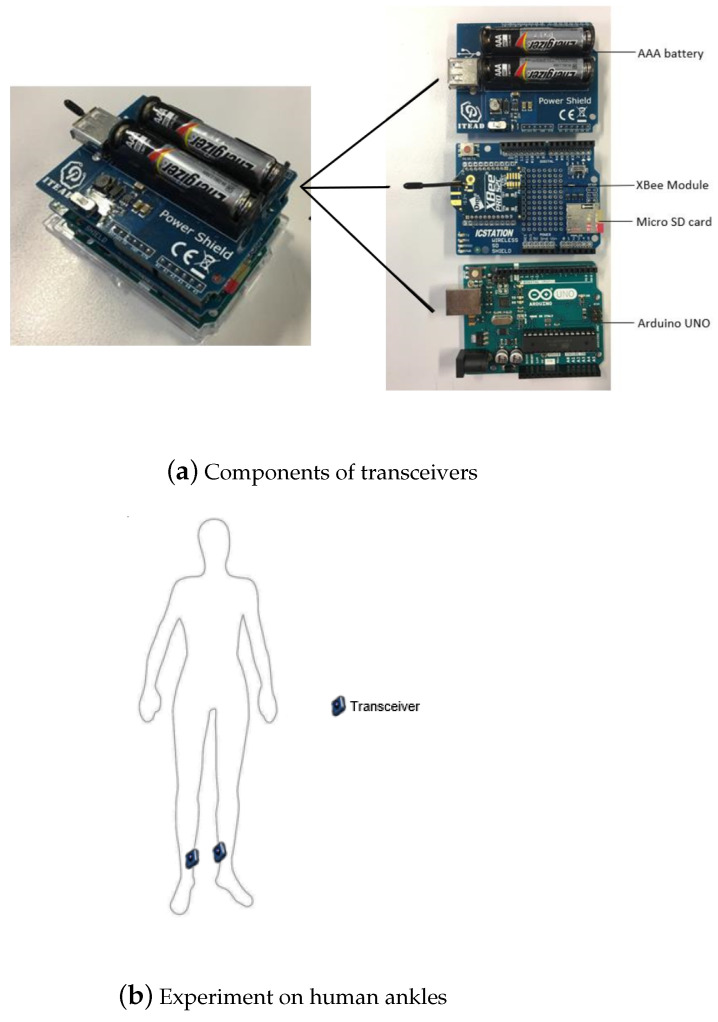
Hardware deployment.

The wireless system works in the 2.4 GHz industrial, scientific and medical (ISM) band, which is one of the carrier frequencies of the IEEE standard for local and metropolitan area networks [27]. The wireless transmitter and receiver are built by using commercial off-shelf components, which are portable, light, affordable, and easy to assemble. They also have little power consumption which enables the on-body usage. One of the Arduino UNO boards works as the coordinator and the other works as the end device. After all the configurations, the Arduino UNO boards, XBee-PRO S2C modules, and micro SD card are assembled. The construction of the transceivers is depicted in Figure 1a. The main purpose of this system is to transmit and receive continuous data packets to/from each other, and a micro SD card is used to save the RSSI values.

In this paper, a point-to-point link with one transmitter and one receiver is considered. Both transceivers are placed at the same height on human ankles as depicted in Figure 1b. The integrated whip antennas on the 2.4 GHz XBee-PRO S2C chips are used. The length of these integrated antennas is shorter than half of the wavelength. Therefore, these antennas are considered as electromagnetically short antennas. As a result, the reactive near field region is determined by

(6) R<λ,

that is, R<12.5 cm. Meanwhile, the far field is determined as

(7) R>2λ,

that is, R>25 cm. Because our aim is to estimate the step length which is typically 0.4 m to 1 m, in this paper, we only consider the far field region. From Equations (1) and (3), distance will be estimated as

(8) $$d=\frac{\lambda \sqrt{10^{\frac{PL_{OA}-\Delta PL}{10}}}}{4\pi },$$

where $PL_{OA}$ is determined from Equation (5).

**Figure 2:**
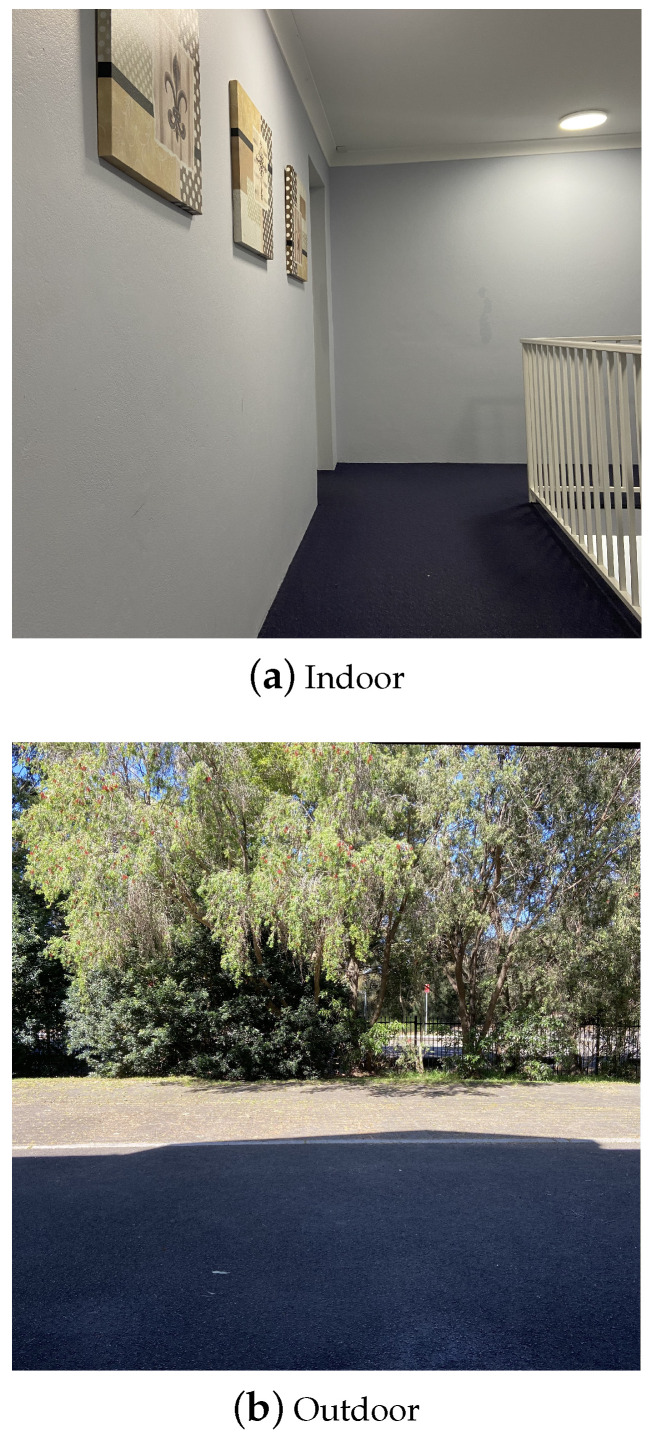
Experimental environment.

The measurements start when the transceivers are placed at a distance of 0.4 m. The transmitter keeps sending sample packets at 9600 bps, with the power level $P_{0}=0$ dBm. In the simulation, the packet length is 4 bytes; thus, 300 frames will be sent each second. In the micro SD card, a three-column text file is recorded. The first two columns are the packet sequence number and the packet time stamp, respectively. The last column is the RSSI values in dB. In this experiment, the hardware Arduino UNO is programmed in a way that, if an erroneous transmission occurs (i.e., the receiver does not receive the packet), a big value of RSSI would be recorded in the file. Thereby, in the later analysis, these erroneous transmissions will be easily detected and omitted. The same measurement procedures at $P_{0}$ are repeated with the distance being increased every 0.1 m, from 0.4 m up to 1 m. We also repeat the experiment for different transmitted power levels and data rates. In this paper, the devices are applied to a female’s body. The measurement on men will be included in the future work. Our experiments were carried out in both indoor and outdoor as shown in Figure 2. The indoor experiments were conducted in a corridor, while the experiment environment for outdoor is a parking area. The experiments are conducted for the static scenario, i.e., the subject under test stands still with different step lengths. We have collected the data for distance from 0.4 m to 1 m and the increment of each step length is 0.1 m. These distances cover the normal step distance for human [28], while guaranteeing the RSSI measurement to be done in the far field of the transmit antenna. Therefore, the static measurement can symbolize the dynamic walking scenario.

## 4. Proposed Path Loss Model

**Figure 3:**
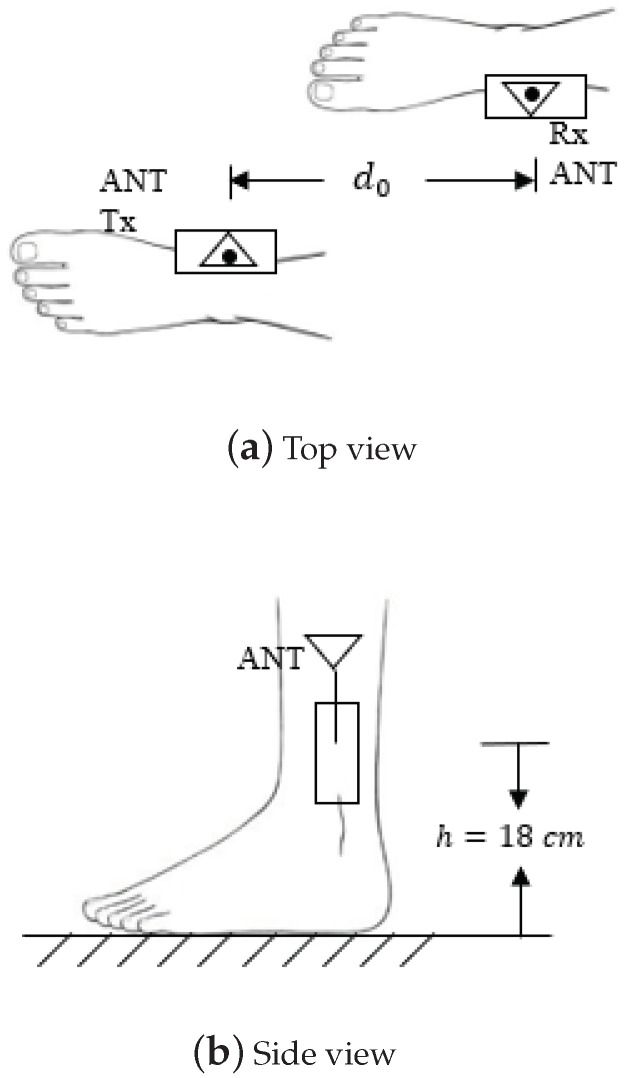
Transceivers attached to the ankles.

**Figure 4:**
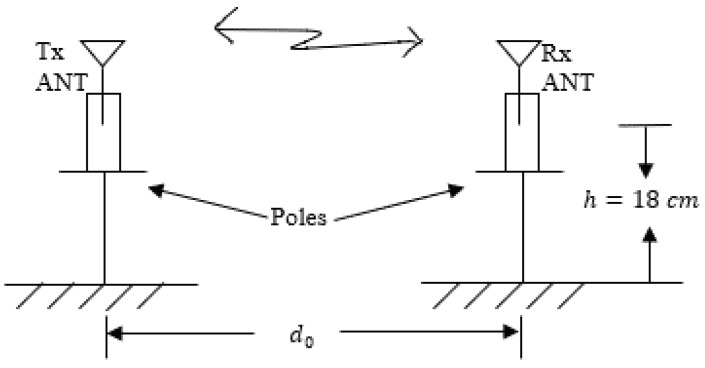
Transceivers attached to the poles.

In this section, an experimental path loss model will be derived. Based on this experimental model, the distance between the transmitter and the receiver can be estimated. Figure 3 and Figure 4 illustrate the experimental schemas where transceivers are attached to the human ankles and poles, respectively. The top view of the on-ankle measurement setup is shown in Figure 3a. The transmitter and the receiver are placed at the medial side of the ankles, while the antennas are pointing upward, orthogonal to the ground. The distance between them is denoted as $d_{0}$. Figure 3b shows the side view of the on-ankle experiment, where the hardware is attached to the ankles at a height of h=18 cm. The schema of the off-body measurement is shown in Figure 4, where the transceivers are attached to the poles at the same height as in the on-ankle experiments.

As mentioned in Section 2, it is conjectured that the path loss between two ankles, denoted as $PL_{OA}$, can be expressed as

$$PL_{OA}=PL_{FS}+\Delta PL,$$

where $PL_{FS}$ is the theoretical free space path loss, and ΔPL is the correction factor. The correction factor can be presented as

(9) $$\Delta PL=PL_{NonL}+PL_{Shad}+PL_{Mul}+PL_{Ins},$$

where $PL_{NonL}$ is the path loss discrepancy due to hardware non-linearity, $PL_{Shad}$ represents the path loss of shadowing effect, $PL_{Mul}$ denotes the path loss caused by multipath propagation, and $PL_{Ins}$ denotes the mismatch loss and insertion loss. We will quantify these path losses in the following, Section 4.1, Section 4.2, Section 4.3 andSection 4.4.

### 4.1. Non-Linearity

Arduino UNO and XBee-PRO S2C are cheap commercial hardware, which may have a certain level of hardware non-linearity. The hardware non-linearity might include the power-dependent non-linearity and the rate-dependent non-linearity.

**Figure 5:**
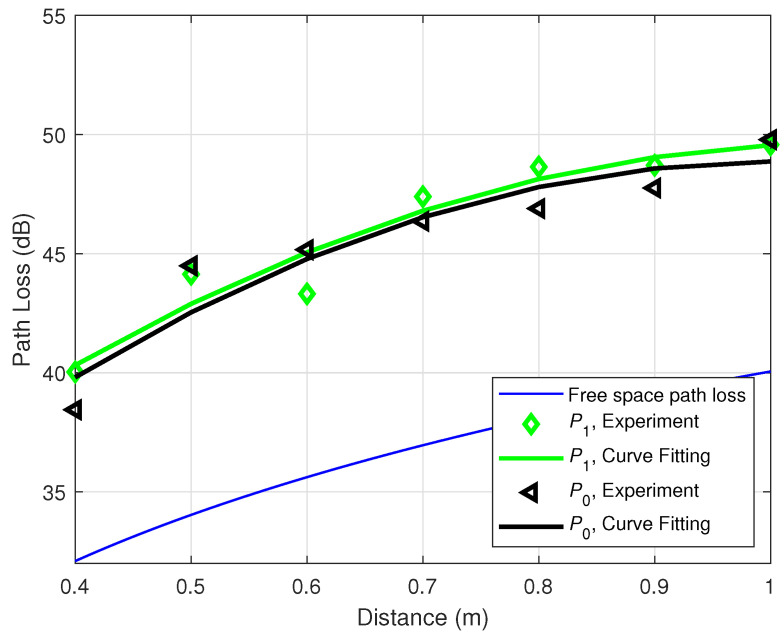
On-ankle path loss for different power levels at the data rate 9600 bps.

The first experiment is conducted under the default data rate of XBee-PRO S2C, which is 9600 bps [29]. Figure 5 demonstrates the relationship between the distance and the path loss between the transmitter and the receiver for different power levels at the same transmission rate. The transmitted power level can be found in Table 2. The blue line at the bottom is the free space path loss calculated by Equation (4), which is used as the reference line to evaluate different hardware configurations. The black triangle markers and the green diamond markers are the average values of the measured experimental path loss at the power levels $P_{0}=0$ dBm and $P_{1}=+12$ dBm, respectively. The corresponding colored solid lines are the experimental fitted curves for these two power levels. From Figure 5, it is clear that there is a little difference between the two path losses corresponding to the two power levels. In theory, the path loss only depends on frequency and distance, while, in practice, the experimental path loss may depend on the transmit power due to the power-dependent non-linearity of the hardware as shown in Figure 5. However, the effect of power-dependent non-linearity to the path loss is small; thus, it will be ignored in Equation (9), i.e., $PL_{NonL}\approx 0$ dB. Figure 5 also shows that the path loss between the two transceivers follows the same trend as that in the free space propagation.

**Figure 6:**
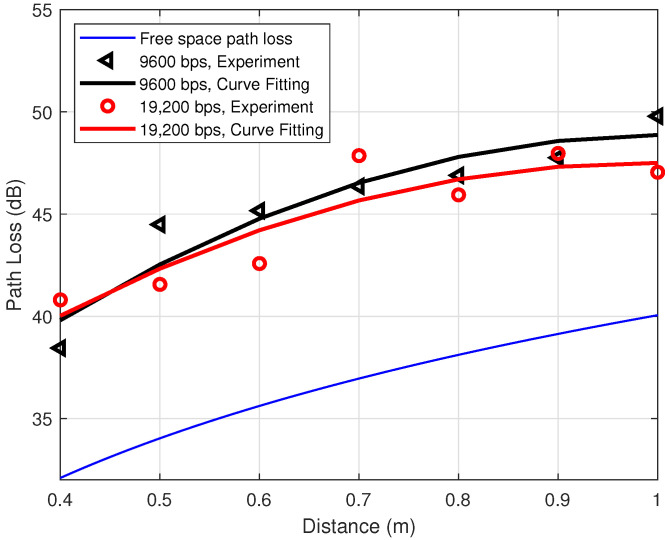
On-ankle path loss for different data rates at the transmitted power $P_{0}$ = 0 dBm.

The transmission rate is another variable in the experiment which may result in the discrepancy between the theoretical and practical path losses. To investigate the influences of data rate on the path loss between two ankles, the transmitted power is fixed at $P_{0}=0$ dBm, so that the path loss is equal to the RSSI (cf. Equation (5)). Figure 6 describes the relationship between the distance and the path loss when the transmission rate is varied. Similarly, the black left-pointing triangles and the red circles are the average values of 50,000 measured experimental path losses at the data rates of 9600 bps and 19,200 bps, respectively. The corresponding colored solid lines are the fitting curves for these two cases. The bottom blue line is the theoretical free space path loss. In theory, the path loss is influenced by distance and frequency only, while, in practice, according to Figure 6, there is also a dependence on the data rate. With an increment of distance, the difference between the two cases of 9600 bps and 19,200 bps increases gradually. Within the step length 0.6 m to 0.8 m of our interest, the discrepancy is around 2 dB. Therefore, the non-linearity due to the transmission rate may contribute to the total correction factor ΔPL by about 2 dB, i.e., $PL_{NonL}\approx 2$ dB.

### 4.2. Shadowing Effect

**Figure 7:**
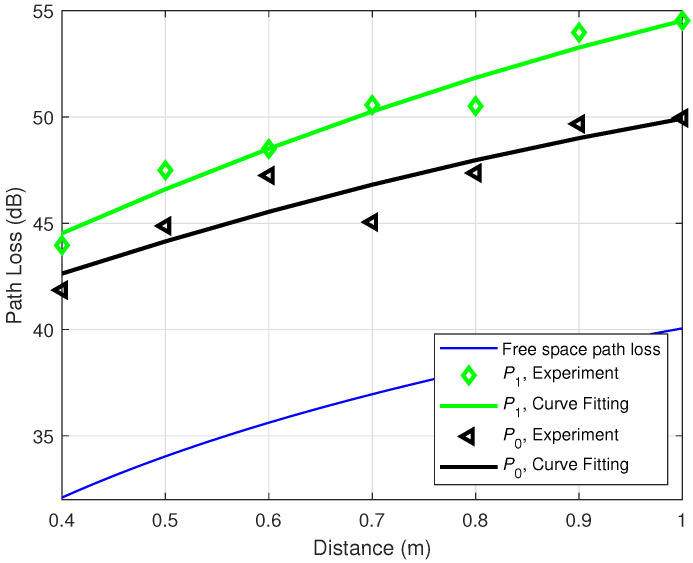
Off-body path loss for different power levels at data rate 9600 bps.

**Figure 8:**
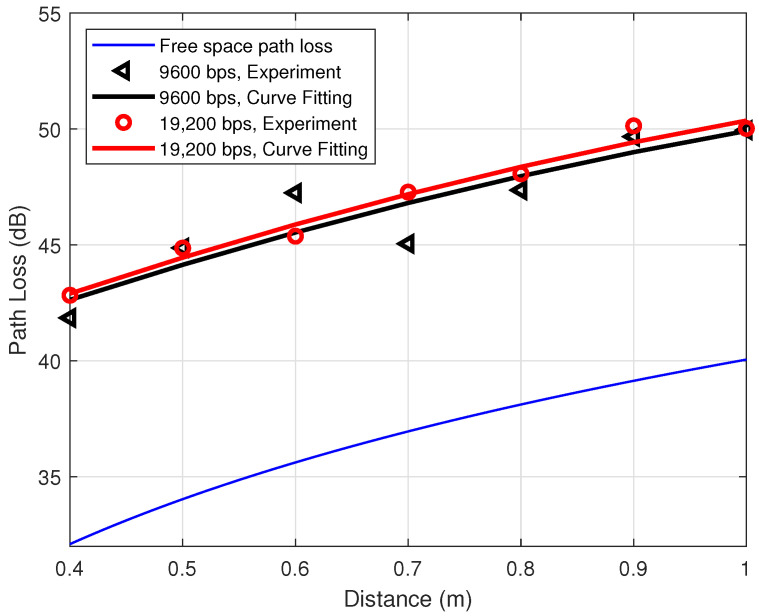
Off-body path loss for different data rates at transmitted power $P_{0}=0$ dBm.

To explore the shadowing effect caused by the human body, experiments are conducted for the off-body situation by removing the transceivers from human ankles (cf. Figure 3a) to two holding poles at the same height *h* of the human ankles as shown in Figure 3b. Other configurations are kept the same as the previous experiments. Figure 7 and Figure 8 are the path loss for the off-body scenarios. More specifically, Figure 7 depicts the experimental path loss at the data rate 9600 bps with different power levels. The left-pointing triangle markers are the experimental data, while the corresponding black curve is the fitting curve, which matches very well with the one in Figure 5. Figure 8 illustrates the experimental path loss data and the fitting curves for the power level $P_{0}=0$ dBm with different data rates. These two curves match well with the black and red curves in Figure 6, especially in the range of interest 0.6–0.8 m. This indicates that the shadowing effect caused by the human body is not significant in our experiment, i.e., $PL_{Shad}\approx 0$ dB. This conclusion is reasonable and supported by our experiment configurations. In particular, in our experiments, the transmitter and receiver are placed on the medial side of the ankles as shown in Figure 3. Thus, there is barely any body tissue between them to cause the shadowing.

### 4.3. Multipath Propagation

**Figure 9:**
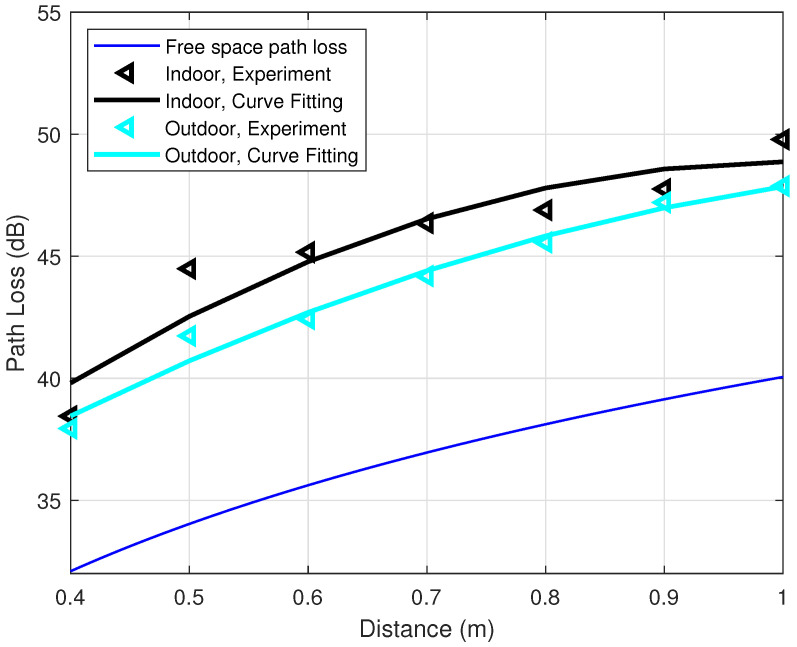
Indoor and outdoor comparison of path loss at transmitted power $P_{0}=0$ dBm, 9600 bps.

The influence of multipath propagation is examined by repeating the experiment with the transceivers attached to the human ankles for the outdoor environment, which is shown in Figure 2b. Figure 9 shows the relationship between the distance and the path loss between two ankles in the indoor and outdoor environments with the same configurations. The transmitted power is 0 dBm and the data rate is 9600 bps. The blue line at the bottom is the theoretical free space path loss calculated by Equation (4). The other two lines on top are the two corresponding curve-fitting lines for indoor and outdoor cases. The black and cyan left-pointing triangles are the average values of the measured path loss for indoor and outdoor, respectively. Figure 9 shows that the cyan curve is persistently below the black curve by 2 dB. This indicates that, for the indoor situation, the multipaths contribute to the path loss correction factor by about 2 dB, i.e., $PL_{Mul}\approx 2$ dB.

### 4.4. Insertion and Mismatch Losses

In this section, both insertion and mismatch losses will be mentioned. Insertion loss is the loss of signal power due to the insertion of a component [30], while the mismatch loss happens due to impedance mismatches and signal reflections. In our experiments, the XBee SD shields are deployed on top of the Arduino UNO boards in order to connect the XBee radio frequency (RF) modules to the Arduino boards as shown in Figure 1a. The insertion and mismatch losses due to hardware, including the XBee modules and the XBee SD shields, are typically 3 dB at each transceiver. This results in a total insertion and mismatch losses of 6 dB, which are unavoidable because of the hardware, i.e., $PL_{Ins}\approx 6$ dB.

### 4.5. Overall Effects of Component Losses

In summary, the influence of the nonlinearity is about 2 dB, the shadowing effect can be ignored, the multipath propagation contributes 2 dB to the total correction factor and the insertion and mismatch losses are around 6 dB. Therefore, the correction factor in Equation (9) can be written as:

(10) $$\begin{array}{cc} \Delta PL(dB) & =PL_{NonL}+PL_{Shad}+PL_{Mul}+PL_{Ins}\\ \; & \approx 2+0+2+6\\ \; & =10. \end{array}$$

As a result, the path loss between the two transceivers on the human’s ankles can be expressed as

(11) $$PL_{OA}\left(dB\right)=PL_{FS}+10.$$

As the experimental path loss model fits very well with the practical path loss in our experiments for the transmitted power $P_{0}=0$ dBm and the data rate of 9600 bps, this configuration is adopted for the distance measurement below. It is worth noting that $P_{0}=0$ dBm and the data rate of 9600 bps are in fact the two default parameters of the Arduino UNO microprocessors.

## 5. Distance Measurement Accuracy

**Figure 10:**
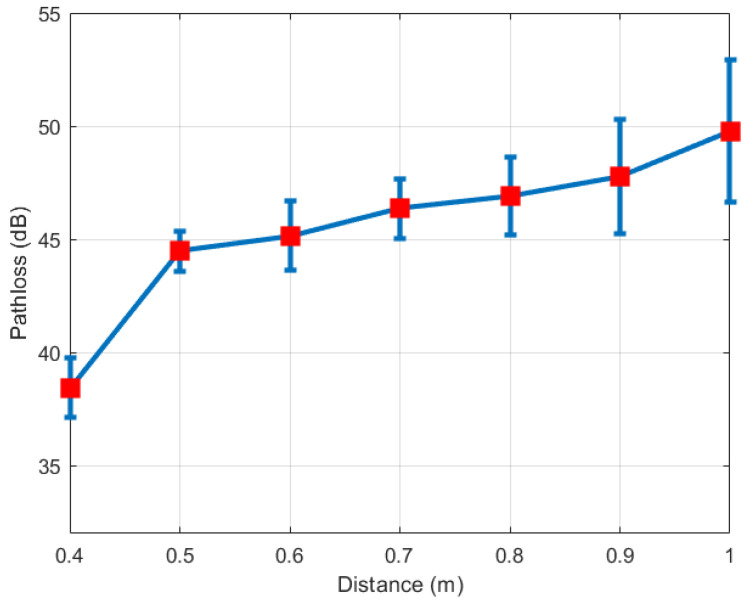
Standard deviation of path loss at $P_{0}=0$ dBm, 9600 bps.

**Figure 11:**
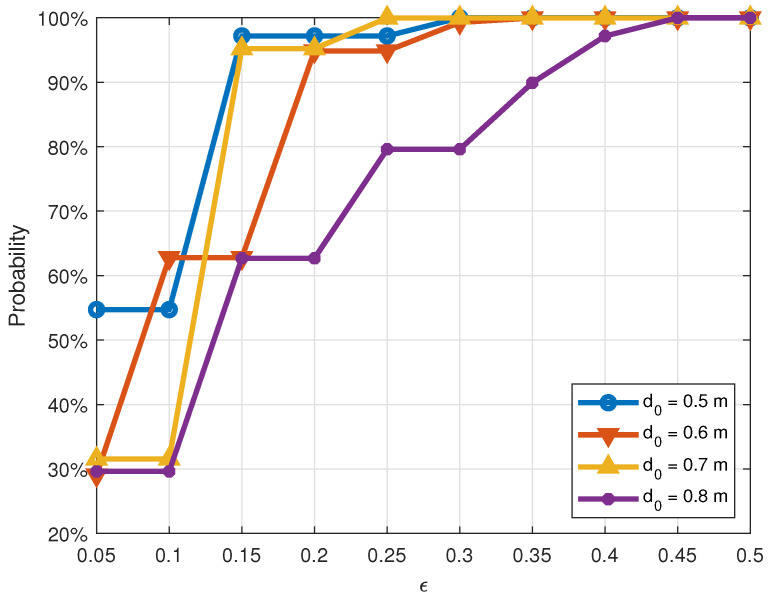
Cumulative distribution function of normalized error ϵ at different distances.

**Figure 12:**
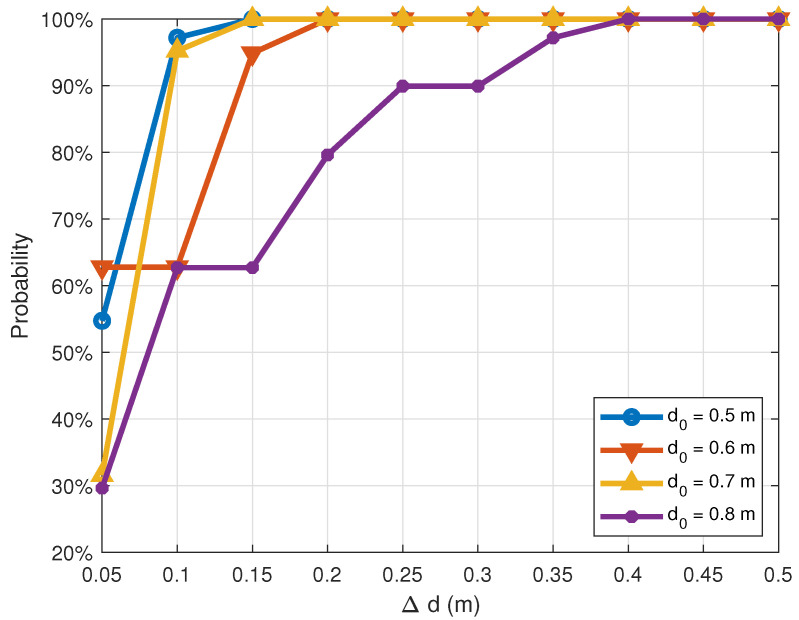
Cumulative distribution function of absolute distance error Δd at different distances.

Figure 10 illustrates the mean and standard deviation of the path loss for different distances at the transmitted power 0 dBm and the default data rate 9600 bps. The red square markers represent the mean values of the on-ankle path losses, while the blue vertical segments present the standard deviation of the measured path loss for each distance. A smaller standard deviation indicates the more consistent measured path loss values, while a bigger standard deviation means the measured path loss values spread over a wider range around the mean values. The mean value and standard deviation value together reflect the accuracy of the path loss measurement. The observation is the accuracy of the path loss measurement generally reduces when distance increases. The measurement accuracy of the path loss value $PL_{OA}$ in turns affects the measurement accuracy of the step length based on the relationship in Equation (8). Therefore, a deduction can be drawn from Figure 10 is the distance measurement accuracy reduces when the real distance increases. This deduction will be confirmed by Figure 11 and Figure 12 mentioned later in this section.

From the measured RSSI values, the distance between the transmitter and the receiver can be estimated based on Equations (5) and (8). The estimated average distance is then calculated by averaging 50,000 realizations. To evaluate the accuracy of the distance measurements, the absolute distance error is defined as

(12) $$\Delta d\left(m\right)=|\bar{d}-d_{0}|,$$

and the normalized distance error ϵ is expressed as

(13) $$\epsilon =\frac{\Delta d}{d_{0}}=\frac{|\bar{d}-d_{0}|}{d_{0}},$$

where $\bar{d}$ and $d_{0}$ are the estimated average distance and the actual distance between the transmitter and the receiver, respectively. From Equation (12), the relation between the estimated average distance $\bar{d}$ and the actual distance $d_{0}$ can be written as $\bar{d}=d_{0}\pm \Delta d$.

Figure 11 and Figure 12 illustrate the cumulative distribution functions of correctly estimating the distance between two transceivers subject to the normalized distance error ϵ the absolute distance error Δd, respectively. From the two figures, the observation is that, in general, the accuracy of distance measurements decreases as the actual distance $d_{0}$ increases. For example, in Figure 11, when the real distance $d_{0}$ is 0.5 m, the probability of estimating correctly this distance with the normalized distance error ϵ not exceeding 0.05 (i.e., Δd≤2.5 cm) is 55%. If this threshold expands to 0.15 (i.e., Δd≤7.5 cm), the probability would be 97%. Meanwhile, these probabilities for the real distance $d_{0}=0.8$ m are only 30% and 63%, respectively. This can be explained as follows: at a shorter distance, the signal is stronger, and the path loss is smaller. Thus, the probability of recovering accurate distances will be higher than that of long distances.

This behavior is also reflected in Figure 11 and Figure 12. Overall, when the distance increases from 0.5 m to 0.8 m, the measurement accuracy is reduced. As shown in Figure 12, when the absolute error Δd=0.1 m, the measurement accuracy of $d_{0}=0.7$ m is 95.21%, which is roughly the same as that of $d_{0}=0.5$ m. When Δd=0.15 m, the accuracy reaches 100% in both cases.

## 6. Conclusions

In this paper, we have developed portable transceivers using Arduino UNO and XBee-PRO S2C. The developed transceivers are used to measure the received signal strength and to analyze the wireless channel between two ankles. We have also derived the experimental path loss model, which is equal to the path loss in free space plus a correction factor of 10 dB. This experimental path loss model is then used to estimate the distance between the transmitter and the receiver attached to the human ankles. The experimental results indicate that the distance can be calculated relatively accurately, despite the cheap hardware devices, with the distance errors of a centimeter scale.

## 7. Limitations and Future Works

In this paper, all the experimental data are collected under a static situation. In the future, we will conduct experiments under different daily activities to estimate the distance between the human body parts. In addition, we have only considered single-antenna transceivers in this work. Multi-antenna transceivers can achieve a higher data rate or throughput, while maintaining a good bit error rate (BER) of the system. Our future works might include the adoption of the multi-antenna systems to increase the accuracy of the localization algorithm. Cooperative communication [25,31,32,33] between sensors on the human body is another aspect that is worth considering to improve the communication reliability and throughput.

## Figures and Tables

**Table 1:** Comparison between various research works focused on the Received Signal Strength Indicator (RSSI) method of distance estimation.

	[12]	[13]	[14]	[18]	[19]	[20]	[21]	[5]	This Paper
Wireless localization	x	x	x	x	x	x	x	x	x
Localization techniques	RSSI	RSSI	RSSI	RSSI	RSSI	RSSI	RSSI + AOA	pAOA + qRSSI	RSSI
Channel model	Not mentioned	Not mentioned	Log-normal shadowing model	An experimental indoor propagation model RSSI= $-(10nlog_{10}d+A)$	Experimental on-body channel model	Log-normal shadowing model with dynamic variance (LNSM-DV)	Not mentioned	Flat Rayleigh fading channel; Log-normal shadowing model	Experimental model $PL_{OA}=$ $PL_{FS}+\Delta PL$
Size of experimental data set (samples)	100	Not mentioned, claim to be small	60	Not mentioned	192,000	100	Not mentioned, claim to be large	N/A	50,000
Hardware	ZigBee with CC2430 chip	Ubiquitous device with CC2420 radio controller	3+ Wi-Fi access points	Bluetooth	Arduino UNO + ZigBee	Micaz nodes with CC2420 radio controller	Bluetooth sensor (mobile phone) and a Bluetooth low energy (BLE) device	N/A	Arduino UNO + ZigBee
Antenna	Omni directional whip antenna	monopole antenna	Omni directional antenna	Directional antennas (e.g., patch antenna) or antenna arrays	Integrated antenna	Dipole antenna	Dipole antenna	Antenna array	Integrated antenna
Shadowing effect	N/A	N/A	x	x (6–8 dB)	x	x	N/A	x	x
Method to estimate the distance	Gauss model (GM)	Maximum- likelihood (ML)	Trilateral localization	Triangulation –least square estimation (LSE)	N/A	Least square (LS)	RSSI-with- Angle-based localization estimation (RALE)	Trilateration+ triangulation	Driven by the measurement- based path loss expression
Nodes	4	0.27 nodes/m${}^{2}$	4	4	3	3	2	Max(p, q)	2
Range	20 m	7.08 m × 10.60 m conference room	8 m × 7 m laboratory	6 m × 8 m classroom	N/A	7 m	360	100 m × 100 m rectangle	1 m
Error	2 m	1.5–2 m	Angle error ±(5°–15°)	1.2 m	N/A	N/A	84%	83%	Centimeter level

**Table 2:** Transmitted power of different settings for XBee-PRO S2C RF modules.

Power Level	Transmitted Power	Notation
0	0 dBm	$P_{0}$
1	+12 dBm	$P_{1}$
2	+14 dBm	$P_{2}$
3	+16 dBm	$P_{3}$
4	+18 dBm	$P_{4}$

## Data Availability

The data presented in this study are available on request from the corresponding author.

## Abbreviations

The following abbreviations are used in this manuscript:

AOA	Angle of Arrival
BER	Bit Error Rate
LNSM	Log-Normal Shadowing Model
LNSM-DV	Log-Normal Shadowing Model with the Dynamic Variance
LOS	Line of Sight
LS	Least Square
PARP	Peak-to-Average Power Ratio
RF	Radio Frequency
RSSI	Received Signal Strength Indicator
VANET	Vehicular Ad Hoc Network
WBAN	Wireless Body Area Network
WSN	Wireless Sensor Network
